# Superiority of Graphene over Polymer Coatings for Prevention of Microbially Induced Corrosion

**DOI:** 10.1038/srep13858

**Published:** 2015-09-09

**Authors:** Ajay Krishnamurthy, Venkataramana Gadhamshetty, Rahul Mukherjee, Bharath Natarajan, Osman Eksik, S. Ali Shojaee, Don A. Lucca, Wencai Ren, Hui-Ming Cheng, Nikhil Koratkar

**Affiliations:** 1 Mechanical, Aerospace and Nuclear Engineering, Rensselaer Polytechnic Institute, 110 8^th^ Street, Troy, New York 12180, USA; 2 Civil and Environmental Engineering, South Dakota School of Mines and Technology, Rapid City, South Dakota 57701, USA; 3 Department of Materials Sciences and Engineering, Rensselaer Polytechnic Institute, 110 8^th^ Street, Troy, New York 12180, USA; 4 Mechanical and Aerospace Engineering, Oklahoma State University, 218 Engineering North, Stillwater, Oklahoma 74078, USA; 5 Shenyang National Lab for Materials Science, Institute of Metal Research, Chinese Academy of Sciences, Shenyang 110016, China

## Abstract

Prevention of microbially induced corrosion (MIC) is of great significance in many environmental applications. Here, we report the use of an ultra-thin, graphene skin (Gr) as a superior anti-MIC coating over two commercial polymeric coatings, Parylene-C (PA) and Polyurethane (PU). We find that Nickel (Ni) dissolution in a corrosion cell with Gr-coated Ni is an order of magnitude lower than that of PA and PU coated electrodes. Electrochemical analysis reveals that the Gr coating offers ~10 and ~100 fold improvement in MIC resistance over PU and PA coatings respectively. This finding is remarkable considering that the Gr coating (1–2 nm) is ~25 and ~4000 times thinner than the PA (40–50 nm), and PU coatings (20–80 μm), respectively. Conventional polymer coatings are either non-conformal when deposited or degrade under the action of microbial processes, while the electro-chemically inert graphene coating is both resistant to microbial attack and is extremely conformal and defect-free. Finally, we provide a brief discussion regarding the effectiveness of as-grown vs. transferred graphene films for anti-MIC applications. While the as-grown graphene films are devoid of major defects, wet transfer of graphene is shown to introduce large scale defects that make it less suitable for the current application.

Literature indicates that the annual costs for corrosion^1^,^2^, including direct and indirect costs, are now approaching $1 trillion which is ~6% of the national GDP of the United States. Studies indicate that microbially induced corrosion (MIC) problems account for ~50% of the total corrosion costs^3^. The MIC problem spans a range of industries including aviation, oil and energy, shipping, and wastewater infrastructure^1^. In fact, MIC is a ubiquitous problem in the natural environment as indigenous microbes are adept at corroding metallic structures under ambient temperatures and neutral pH conditions^4^,^5^,^6^. MIC is caused by a genetically diverse set of microbes that exist in harmony (encapsulating themselves in a matrix of self-excreted slimy exopolymeric substance), and form a robust biological film (i.e. biofilm)^3^,^5^,^7^. The biofilm accelerates the corrosion process^8^ by modifying the chemistry of the protective metal oxide passivation layers^8^. Prevention of MIC is cumbersome as it requires constant detection and monitoring of microbial populations. Moreover, physical methods for eradication of biofilms (i.e. flushing) are energy-intensive and may in fact aggravate corrosion by dislodging oxide layers on the metal surfaces^5^.

Metal coatings and alloys have been commercially^6^ used to combat corrosion in abiotic environments. However, when translated to a biotic environment their effectiveness is reduced due to aggressive microbial activity. Further, they suffer from inherent disadvantages such as environmental regulations that prohibit their use for corrosion applications (e.g. Cr)^3^,^7^,^9^,^10^. Polymer coatings (both natural and artificial) have also been used as an effective barrier for corrosion applications but can suffer from poor adhesion to the base materials and undergo rapid microbial degradation^11^,^12^,^13^,^14^,^15^. It has been reported that over time, pin-hole defects induced by microbial activity in polymer coatings grow in size, attract aggressive ions onto metallic surfaces, thereby further accelerating the electrochemical corrosion process^16^. Moreover, the typical thickness of commercial polymeric coatings^17^ disrupts the functionality (e.g. electrical and thermal conductivity) and dimensional tolerances of target metals.

Graphene (Gr), a two-dimensional sheet of sp^2^ bonded carbon atoms, can be employed as an ultra-thin corrosion-resistant coating, as it is mechanically robust, flexible, chemically inert, thermally and electrically conductive, and can form an impermeable barrier^18^,^19^,^20^,^21^,^22^,^23^. Further, ultra-thin graphene coatings can be applied without negatively impacting the functionality (e.g. electrical, thermal conductivity etc.) and dimensions of the underlying metal. Such graphene coatings have been recently demonstrated as corrosion-resistant coatings for metals (e.g. Ni, Cu, Fe, and steel alloys) under abiotic environments^24^,^25^,^26^. However, these studies were based on relatively short time scales (minutes to hours). Recently, two studies have provided some very interesting observations on the failure of graphene coatings on copper substrates under abiotic conditions^27^,^28^. The reason for coating failure was attributed to mass transport through the nanoscale defects present on the graphene sheet, which can be reduced significantly by the use of few-layer graphene^29^. Further, it has been shown that defect plugging (using passive Al_2_O_3_ nanoparticles) caused a significant improvement in the corrosion resistance of monolayer graphene^29^. In our recent study, we found that 3–4 layer graphene films deposited by chemical vapor deposition (CVD) offer long-term resistance (~2400 h) to bimetallic corrosion of Ni, especially under microbial conditions^30^. In this work, we compare the MIC resistance of graphene to two widely used polymer coatings. In particular parylene (PA) is one of the most popular barrier coatings used by industry as it has excellent mechanical properties and provides pin-hole free coatings. Polyurethane (PU) is also widely used to protect surfaces. A detailed electrochemical analysis reveals that the graphene coating offers ~10-fold improvement in MIC resistance compared to PU and ~100-fold compared to PA. This finding is remarkable considering that the average thickness of the graphene coating (1–2 nm) is ~25-fold smaller than PA (40–50 nm), and ~4000-fold lower than the PU coating (20–80 μm). Post-mortem analysis reveals that graphene is highly resistant to microbial attack as compared to the polymer coatings. We perform detailed microbial analysis to comprehend the success of graphene coatings and the failure of polymer coatings. We also compare as-grown vs. transferred graphene films and show that transferred films are far more defective than as-grown ones. In addition, we explore the effect of number of graphene layers and show that few-layered (as-grown) graphene films offer by far the most defect-free surfaces and are therefore the best suited for microbial corrosion resistance applications. Since many of graphene’s fascinating properties strongly depend on the concentration and nature of defects, the defect analysis presented in this study may find broad use in several graphene based applications.

## Results and Discussions

The objective of this study was to investigate the effectiveness of nano-scale graphene coatings to prevent MIC of the immersed, metallic structure under galvanic conditions. More specifically, the MIC resistance of Ni was compared in presence of three different coatings: i) parylene-C coating on Ni (PA/Ni), ii) polyurethane coating on Ni (PU/Ni) and iii) graphene coating on Ni (Gr/Ni). A Ni foam^30^ was used as the substrate for all three coatings.

**Figure 1:**
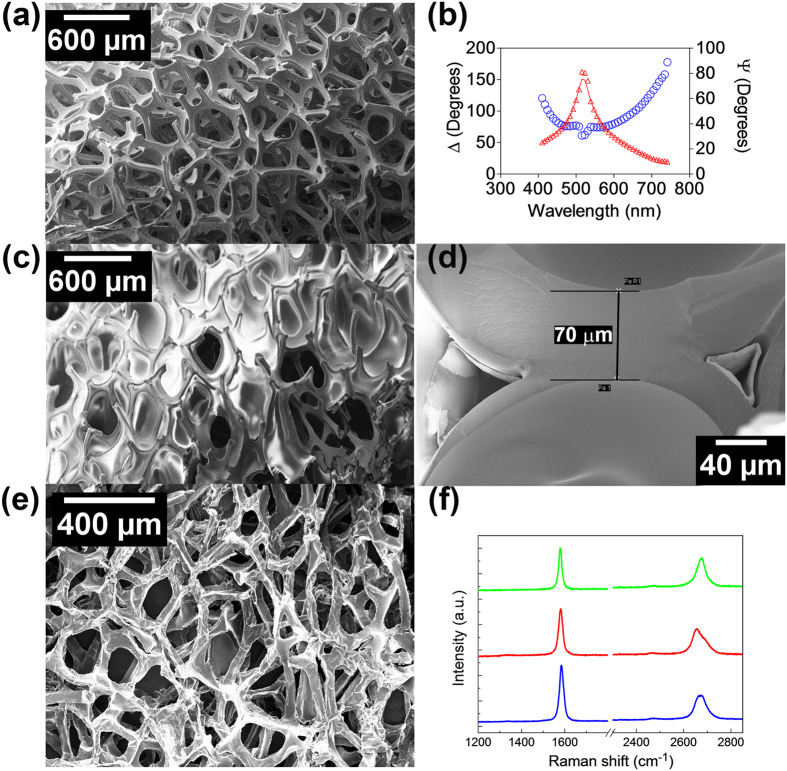
Dimensional characteristics of three coatings on Nickel foam surfaces: (**a**) Parylene (PA) coated Nickel, (**b**) Ellipsometry shows ~46 nm PA coating, (**c**) Polyurethane (PU) coated Nickel, (**d**) SEM image showing thickness of PU coating of 20–80 microns, (**e**) Conformal coating of graphene film on a Ni foam and (**f**) Raman spectra of Gr/Ni foam at three different locations indicating that the graphene film is on average comprised of few-layer (~3–4 layer) graphene.

**Figure 2:**
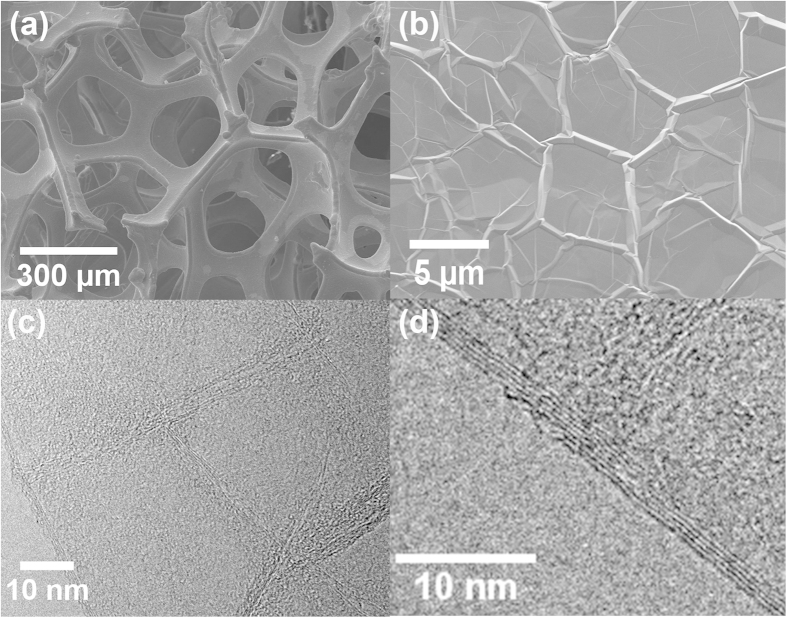
(**a**) Low magnification scanning electron microscopy (SEM) image of Ni foam, (**b**) high magnification SEM image, showing the wrinkled graphene topography on the Ni foam surface, (**c**) low magnification transmission electron microscopy (TEM) image of few-layer graphene on Ni, (**d**) high magnification TEM image showing 4 layers of graphene on the Ni surface.

Chemical vapor deposition (CVD- see methods section) was used to obtain a uniform coating of PA–C on the Ni surface (Fig. 1a). The thickness of the coating was determined using ellipsometry. The ratios of the amplitude (ψ), and phase changes of the p and s components (Δ) of polarized light, are shown in (Fig. 1b). Using the Cauchy coefficients (A = 1.58 and B = 0.012)^31^, the average thickness of the PA film was estimated to be ~46.1 nm. The PU films were deposited using standard spray coating (see methods section). As shown in Fig. 1c,d, scanning electron microscopy (SEM) imaging was used to determine the thickness of PU/Ni in the range of 20–80 μm. The graphene films (Fig. 1e) were deposited on the Ni foam using CVD (see Methods section) as shown in our previous study^30^. From the Raman analysis of the Gr-Ni sample (Fig. 1f), the I(2D)/I(G) ratio^30^,^31^,^32^ indicates a fingerprint of few-layered (3–4 layers) graphene coatings. This was also confirmed by high-resolution scanning electron microscopy (Fig. 2a,b) and transmission electron microscopy (Fig. 2c,d) of the graphene coating. Further, the absence of the Raman D band (~1350 cm^−1^) in Fig. 1f confirms the low density of defects in the Gr film^32^.

**Figure 3:**
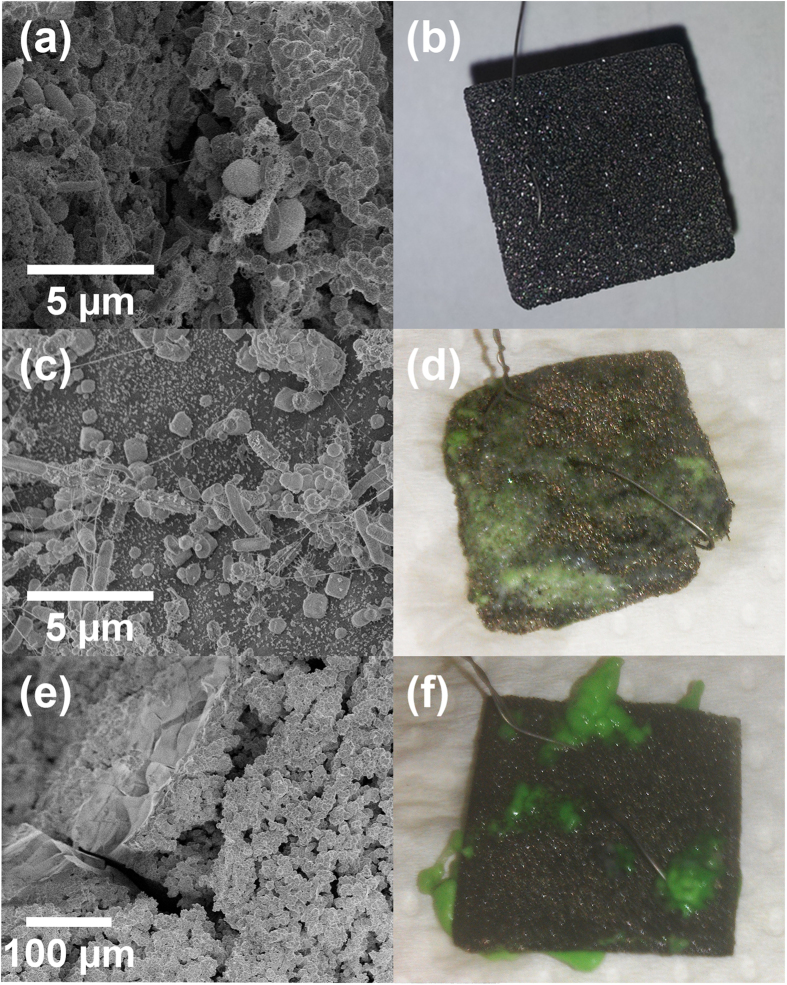
(**a**) SEM image of biofilm on Gr/Ni, (**b**) MIC-resistant Gr/Ni anode after 30 days of MIC testing, (**c**) SEM image of biofilm on PA/Ni, (**d**) Corroded Ni/PA anode after 30 days of MIC experiment, (**e**) SEM image of biofilm on PU/Ni and (**f**) Corroded Ni/PU anode after 30 days of MIC experiment.

The corrosion cells (Fig. S1, Table S1–S2, supporting information) were operated in a fed-batch mode for 30 days to promote the MIC conditions that induce bimetallic corrosion of the Ni anode. The SEM micrographs (Fig. 3a,c,e) provide a clear evidence for the presence of biofilm on the surfaces of Gr/Ni, PA/Ni, and PU/Ni. Further, visual examination of PA/Ni (Fig. 3d) and PU/Ni (Fig. 3f) indicates the presence of green corrosion by-products (e.g. Ni (II) compounds). The edges of the PA/Ni were found to be corroded possibly due to direct (metal-microbe interaction) and indirect action of microbes (intermediate metabolites such as volatile fatty acids)^10^. Figure 3 suggests that the anti-MIC behavior of PU/Ni is slightly better than that of PA/Ni. The most important observation is that the graphene coating was able to preserve the underlying Ni material (Fig. 3b) even after continuous exposure to the MIC environment for 30 days.

### Electrochemical studies

**Figure 4:**
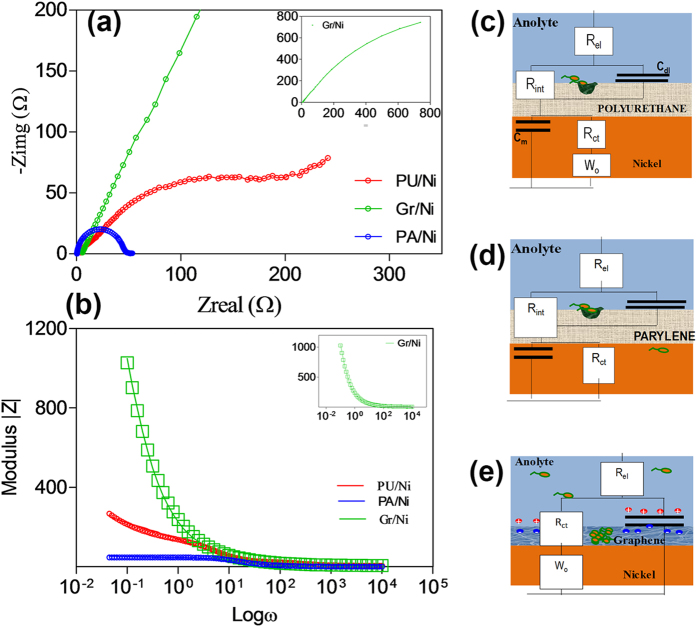
(**a**) Nyquist Plots for the three coatings, (**b**) Bode Modulus plots for the three coatings, (**c**) Equivalent circuit for PU/Ni, (**d**) Equivalent circuit for PA/Ni and (**e**) Equivalent circuit for Gr/Ni.

The Nyquist plots (Fig. 4a) shows the anodic impedance (sum of polarization resistance and electrolyte resistance) of the three corrosion cells in a complex-impedance-plane. The asymptote in the low-frequency region of the Bode plots (Fig. 4b) represents the total impedance to MIC corrosion of Nickel. Among the three coatings, graphene coating (Gr/Ni) offers the highest anodic impedance followed by PU/Ni and PA/Ni (Fig. 4a,b). Another important finding can be inferred from the locus of the points for PU/Ni and PA/Ni in Fig. 4a; both the curves do not trace a true semicircle implying that the anodic impedance response does not correspond to a single activation-energy controlled process.

Figure 4a,b indicates that dissimilar anodic processes occur in Gr/Ni, PU/Ni, and PA/Ni. Hence, we used three different equivalent circuits (Fig. 4c–e) that are descriptive of the three coatings varying in thickness and anti-MIC properties. In the PU/Ni, the thickness of PU coating ranged from 20 to 80 μm, thereby the Ni foam can be considered to be embedded in the aggregate mixture of polymer matrix and biofilm^33^. The aggregate film on the Ni surface is porous and the electrochemical reactions are likely to occur only on the exposed surfaces at the end of a pore (Fig. 4c). At the interface located at the end of the pore, the corresponding corrosion impedance is the parallel combination of charge transfer resistance (R_ct_) and interfacial capacitance (C_m_). The seepage of anolyte through the PU coating is responsible for the charge transfer reactions associated with the corrosion process. In order to model diffusion limitations of corrosion by-products through the porous electrode, a Warburg element (W_O_) is added in series to the corrosion impedance. Within the pore length, the electrolyte resistance is R_int_, and the insulating part of the coating can be considered to be a capacitor C_dl_ which is in parallel with the impedance in the pore (Fig. 4c). The electrolyte resistance (R_el_) is added in series with the previous impedance.

The thickness of the parylene coating in the PA/Ni system is ~3 orders of magnitude smaller than that of PU in PU/Ni. We therefore used an alternate equivalent circuit model based of the anolyte resistance (R_el_), the anolyte/PA interfacial impedance and PA/Ni interfacial impedance (Fig. 4d). At the anolyte/PA interface, the defects and the pores in the PA coating enable the anolyte to access the underlying nickel surface, and such impedance is modeled as a pore resistance (R_int_). Similarly, the interfacial capacitance at the anolyte/PU interface is modeled as C_dl_. The charge transfer reactions associated with Ni corrosion at the anolyte/PA interface were incorporated as a parallel combination of charge transfer resistance (R_ct_) and interfacial capacitance (C_int_)^34^,^35^.

**Figure 5:**
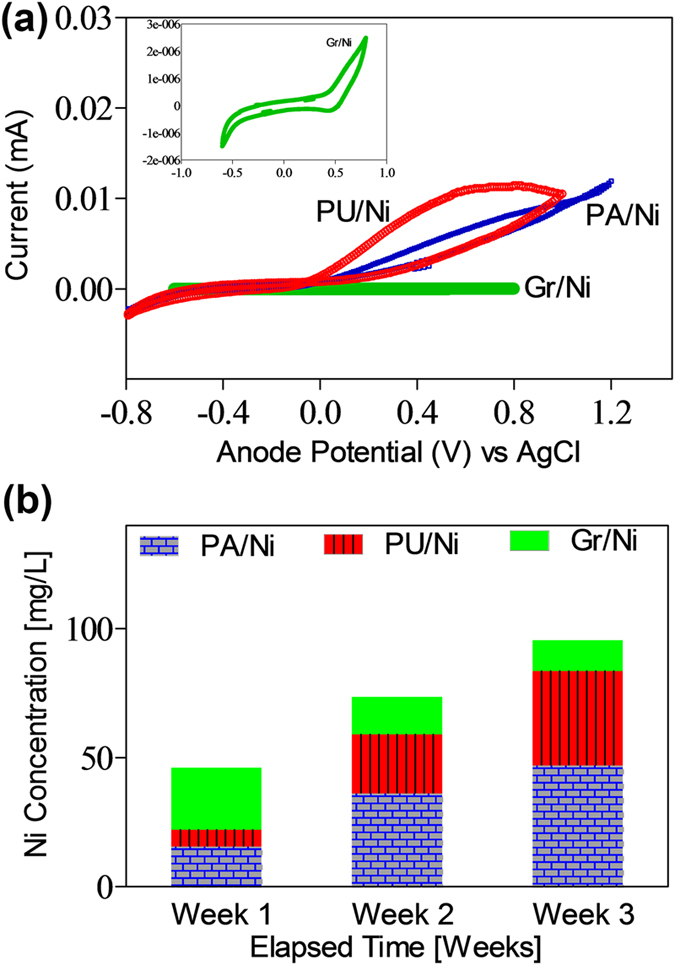
(**a**) Cyclic Voltammograms for the three coatings in corrosion cells, (**b**) Soluble nickel concentration in the anolyte for the three corrosion cells. Note: Corrosion cells were operated in a fed-batch mode; temporal graph for soluble Ni will not follow a linear pattern.

We modeled MIC of Gr/Ni as a modified Randles equivalent circuit (Fig. 4e)^30^,^36^. The circuit fitting analysis revealed that the R_ct_ of Gr/Ni (~35.8 kΩ.cm^2^) was ~10-fold higher than that of PU/Ni (~1.57 kΩ.cm^2^), and ~100-fold higher than that of PA/Ni (~0.38 kΩ.cm^2^) (Details in Table S3, supporting information). We conclude that the graphene coating offers superior MIC-corrosion resistance as compared to PA and PU coatings. The cyclic voltammetry (CV) results also corroborate that graphene provides an inert environment that suppresses Ni corrosion (Fig. 5a). The PU/Ni and PA/Ni registered a higher range of electrochemical current compared to that of Gr/Ni. The maximum current in Gr/Ni was at least 10,000 fold lower than that of PU/Ni. A broad shoulder was observed in the PU/Ni and the PA/Ni responses between 0 and 0.8 V (vs. Ag/AgCl). This denotes a redox reaction occurring on the Ni surface. In comparison, the Gr anode response was devoid of significant oxidation or reduction peaks.

### Nickel dissolution

Figure 5b shows the Ni concentrations in the spent-anolyte at end of three different fed-batch cycles in the three corrosion cells. The concentration of Ni^2+^ in both the PA/Ni and PU/Ni cells rises significantly during the course of three weeks. The corrosion rates during Week 1 were ~2.22 mg/L/day and ~0.915 mg/L/day in PA/Ni and PU/Ni, respectively; in week 3, the rates reached as high as ~6.708 mg/L/day and ~5.229 mg/L/day, respectively. Further, the Ni concentration in the cells with polymer coatings (week 2: PU/Ni ~22.89 mg/l, PA/Ni ~36.08 mg/l, week 3: PU/Ni ~36.604 mg/l, PA/Ni ~46.962 mg/l) increased linearly. In Gr/Ni, the corrosion rates remained in the 1.5–3 mg/L/day range in all the three cycles. The cell with Gr/Ni demonstrated an improvement in the corrosion resistance (week 2: ~14.663 mg/l, week 3: ~11.938 mg/l). The higher values of Ni concentrations in PU/Ni and PA/Ni cells during subsequent weeks denote an eventual failure of the polymer coatings. The lower corrosion rates in the Gr/Ni are likely due to a combination of the following: i) minimal defects in the Gr coating compared to PA and PU coatings, ii) limited diffusion of corrosion by-products from the Ni to the bulk liquid due to restricted access through the few-layered Gr coating, and iii) adhesion of polysaccharides and insoluble biomass to the hydrophobic graphene surface which plugs the few defect sites that may exist on the Gr film surface.

**Figure 6:**
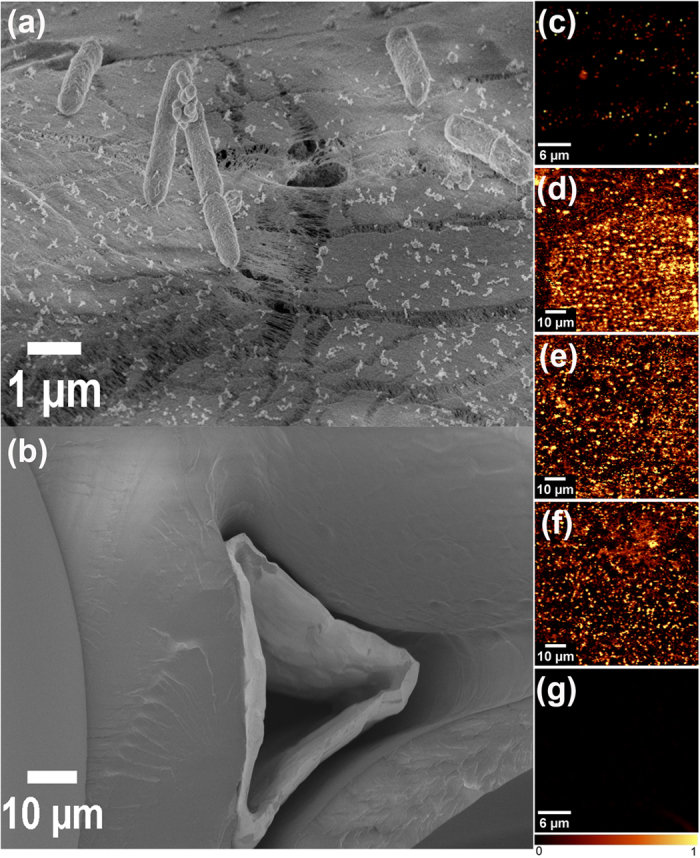
Coating failure by (**a**) Localized tears in PA/Ni electrode, (**b**) Non-conformity and poor adhesion in PU/Ni electrode, Raman mapping of (**c**) monolayer graphene grown on copper (bright spots indicate point defects), (**d**) monolayer graphene transferred onto SiO_2_, (**e**) bilayer graphene transferred onto SiO_2_, (**f**) trilayer graphene transferred onto SiO_2_, (**g**) Few-layer graphene grown *in-situ* on the Nickel Foam by chemical vapor deposition. This as-grown few-layered graphene film is relatively free of defects and provides excellent barrier protection. The scale bars in (**c**) and (**g**) are 6 μm and in (**d**–**f**) are 10 μm.

Scanning electron microscopy (SEM) was used to observe the extent of corrosion-induced debilitation of the PA/Ni and PU/Ni surfaces. We found micron-length tears on the PU/Ni surfaces (Fig. 6a) while the PU/Ni surfaces exhibited poor conformity and poor adhesion to the Ni surface (Fig. 6b). The microbially induced degradation and tearing of the PU coating that we report here is consistent with what has been reported in the literature^37^,^38^,^39^,^40^. The micron-scale tears on the PU/Ni surface lead to i) enhanced charge transfer associated with Ni corrosion, and ii) increased diffusion of intermediate metabolic byproducts (e.g. acetic acid resulting from glucose fermentation) on the Ni surface. Figure 6b indicates the non-conformal nature of the PU coating, and the vacancies at the PU/Ni interface which expose the Ni surface to microbial corrosion. Figure 6a,b justify our observations on the gradual increase in Ni corrosion rates with time (high Ni corrosion rates for PA/Ni and PU/Ni appeared during the later stages of corrosion-cell operation). In comparison, the Gr coating, which is extremely conformal, provides effective barrier protection, and restricts the microbes from accessing the Ni surface, thereby supporting the observed low corrosion rates of the Gr/Ni system shown in Fig. 5b.

### Defect analysis of graphene coatings

On having established that CVD-grown (few-layer) graphene offers superior resistance to MIC, we sought to characterize the defectiveness of the as-grown graphene coating when compared to graphene films that are transferred (wet-transfer^19^) onto surfaces that are incompatible with CVD growth. For this purpose, monolayer graphene, grown *in-situ* on a copper foil by CVD, was transferred layer-by-layer onto SiO_2_ using wet chemistry based transfer methods^19^. Confocal Raman spectroscopy maps of the defect intensity ratio (I(D)/I(G)) were generated for monolayer graphene grown on Cu (~30 μm × 30 μm) (Fig. 6c) and monolayer (Fig. 6d), bilayer (Fig. 6e) and trilayer (Fig. 6f) transferred graphene sheets on SiO_2_ (~80 μm × 80 μm).

The mapping in Fig. 6c shows point defects (bright spots) on the Gr/Cu surface, which can act as the initiation points for corrosion as observed by Schriver *et al.* and Zhou *et al.*^27^,^28^. The Raman maps for the transferred samples (Fig. 6d–f) show an increased presence of defective regions in comparison to the as-grown monolayer graphene (Fig. 6c). These results suggest that the transfer process induces a large number of defects in the graphene films. The overall I(D)/I(G) defect intensity ratio was calculated for samples (Fig. S2, supporting information) and was found to be about an order of magnitude lower for the few-layer graphene grown *in-situ* on Ni (Gr/Ni), as compared to the Gr films transferred onto SiO_2_ and half the value for monolayer Gr as-grown on Cu foil. This is visually represented in the Raman map for the few-layer graphene grown on Ni foam (Gr/Ni- Fig. 6g) used in our testing (Figs 3, 4, 5). Note that the Raman map in Fig. 6g is completely devoid of any bright spots (i.e. defects). Scanning electron microscopy (SEM) images for these samples indicate large scale wrinkles in the monolayer graphene transferred onto SiO_2_ and a notable reduction in the wrinkle density observed for two and three layer transferred graphene samples (Fig. S3, supporting information).

Note that the highest defect intensity ratio (Fig. S2, supporting information) recorded for the monolayer graphene (post-transfer) can be attributed to strong adhesion between SiO_2_ and the graphene layer^41^. On adding further graphene layers, the weak interactions between the similar graphene layers allows the freshly transferred graphene sheet to slide more easily on top of the previous sheet, thereby reducing stress build-up and defect generation during the transfer process. However the few-layer graphene films that are as-grown on the metal surface are by far the least defective (see Fig. 6g and Supplementary Fig. S2) indicating that it is essential to deposit the graphene ‘*in-situ*’ using bottom-up growth techniques such as chemical vapor deposition. Wet-chemistry based transfer methods^19^ to deposit graphene coatings are unlikely to yield pin-hole free coatings. These results indicate that it is crucial to directly grow few-layered graphene films on the surfaces to be passivated rather than post-synthesis transfer onto the surface.

### Microbial phylogenetic analysis and mechanism for superior corrosion resistance of graphene.

**Figure 7:**
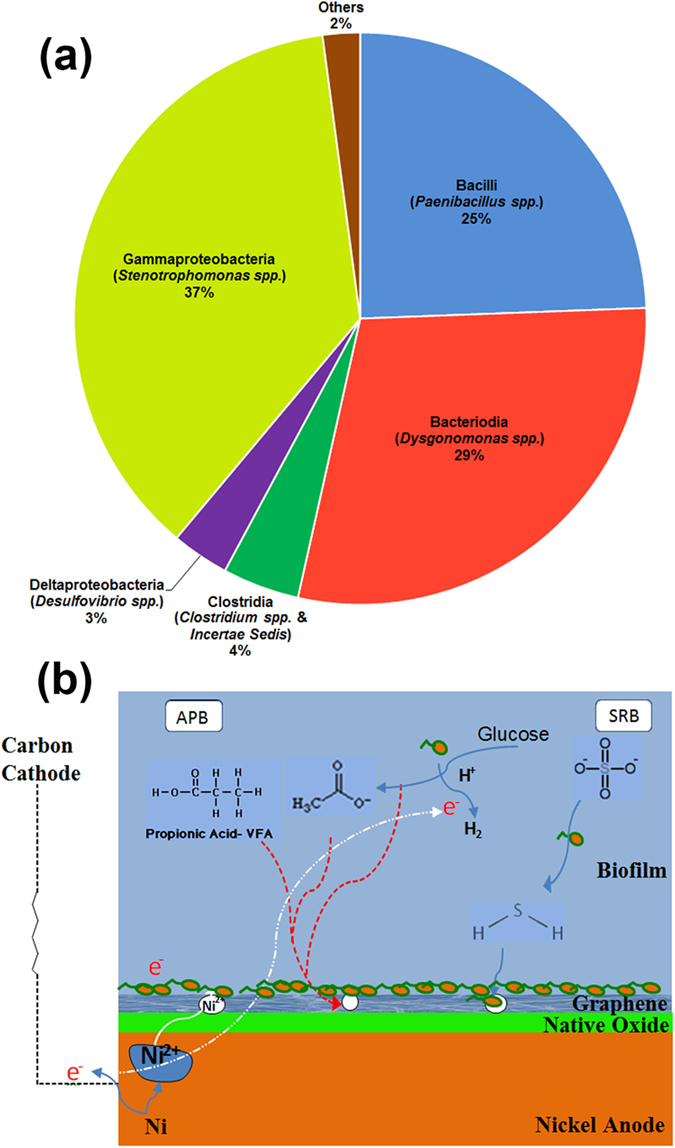
(**a**) Microbial community analysis on corroded nickel surface, (**b**) Proposed mechanism for superior corrosion resistance in the presence of multilayer graphene coating.

Corrosion characteristics are determined by the corroding and the corroded species. In order to better understand the interactions between the microbial community and the coated nickel surfaces, a phylogenetic microbial community analysis was carried out and its results are presented in this section (Fig. 7a). The anaerobic conditions prevalent in the corrosion cell discourage the growth of aerobic microbial communities, thereby promoting anaerobic fermentation and acid production which accelerate the MIC process. Literature^42^,^43^,^44^ indicates that *Stenotrophomonas spp.*, previously isolated from various environments including corroded metal surfaces are capable of degrading xylene-based compounds. These studies suggest that the *Stenotrophomonas spp.* may be responsible for the tears observed on parylene-coated nickel surface. Coupled with the acidic environment caused by the presence of *Dysognomonas spp*.^45^*, Desulfovibrio spp.*^46^*, Clostridium spp.*^47^,^48^, there is rapid degradation of polymer coatings and the nickel foam surface after biofilm formation. Figure 7b depicts the participating microbial and electrochemical reactions that influence the MIC of the Ni surface. The Ni surface is hypothesized to interact with the native Ni oxide, biofilm, the bulk anolyte, and the protective coating. In the absence of coatings, the bare Ni anode undergoes MIC due to the direct and indirect influence of anaerobic bacteria such as acid producing (APB) and sulfur reducing bacteria (SRB)^46^. It should be noted that the nutrients (e.g. glucose) in the anolyte support the growth of APB that secrete organic acids (e.g. acetate)^49^,^50^ (Fig. 7a), and accelerate MIC of Ni surface. Finally, the SRB accelerate MIC of Ni surface^5^,^46^,^51^ in the following sequence: i) SRB reduce sulfur compounds to HS^−^ ions ii) sulfide-oxidizing bacteria convert HS^−^ to sulfate that is quickly oxidized to sulfuric acid and abruptly decrease the local pH (<1.0) to enhance the Ni corrosion.

In our experiments, we observed a change of the anolyte color from yellow to black within 48 hours of initial operation (Fig. S4, supporting information) which can be attributed to sulfide compounds (e.g. FeS) produced by SRB^50^,^51^. In order to confirm the presence of sulfur precipitates on Nickel surface, we operated two different corrosion cells for 800 hours using: i) bare Ni anode (abiotic cell) and ii) bare Ni anode with microbes (biotic cell). The Ni anodes in abiotic and biotic cells were then subjected to a post-mortem analysis using X-ray photoelectron spectroscopy (XPS). As shown in Fig. S5, the percentage of sulfur precipitate (%) on the Ni anode in the biotic cell accumulated to nearly 1.2%, while that in the abiotic cell was negligible (<0.1%). The absence of major cations on the Ni anode suggests that the sulfur atoms are bonded to the surface of the Ni anode.

We observed a declining trend in the temporal values of both i) corrosion current and ii) Ni^2+^ concentration (Fig. 5b) in the Gr/Ni cell. This can be explained on the basis of the time required for the progressive maturation of biofilm growth on the Gr/Ni anode surfaces. The high conductivity of Gr provides ideal conditions for the growth of anode-reducing bacteria at the interface of the Gr/Ni/anolyte. The typical biofilm thickness (~500 nm)^52^,^53^,^54^ is 250–500 fold thicker than that of the graphene film (1–2 nm), and as such, any point defects that may be present on the graphene film surface are likely plugged with non-conductive polysaccharides and microbial debris in the biofilm. Studies find that the H_2_^49^ gas typically involved in cathodic reactions of MICs^46^,^51^ cannot penetrate through the defect-free graphene layer^23^,^55^,^56^, and such conditions will increase the impedance to participating cathodic reactions. Confocal Raman spectroscopy also confirms that the defect intensity ratios (I(D)/I(G)), averaged over a ~30 μm × 30 μm area, were found to be extremely low for the Gr coating (Fig. S2, supporting information). As discussed in the previous section, Fig. 6g shows a typical I(D)/I(G) map for Gr/Ni; the dark regions in the map are the defect free regions with small or negligible I(D)/I(G) values. The Raman mapping results in Fig. 6g confirm that the few-layered Gr coating in the Gr/Ni sample provides a relatively defect-free barrier to combat MIC. The near absence of point defects and their plugging by the biofilm, are hypothesized to cause the large impedance in the form of diffusion limitations to both charged and uncharged species, and thus minimal MIC action. This graphene barrier coating with low defect density also minimizes the formation of crevices, concentration cells, and corrosion pits on the Ni surface. In addition, the few-layered graphene is impermeable to acids and thereby protects the Ni anode from typical under-deposit acid attack encountered in corrosive environments.

## Conclusion

Graphene coatings have not been explored in detail for corrosion prevention in biotic environments. The current study shows that graphene coatings outperforms its polymer counterparts (parylene C and polyurethane) by offering 1 and 2 orders of magnitude better impedance against microbial corrosion. The shortcomings associated with defect based corrosion of graphene coatings in abiotic environments has been countered in this study by the use of 3–4 layer graphene films that are grown *in-situ* with minimimal defect density. Further, the few defects that may be present on the surface can be plugged by biofilms through the production of non-conductive polymeric debris. On exposure to similar harsh microbial environments, parylene C failed by extensive microbial degradation and polyurethane due to its non-conformal adhesion to the metallic surface. In order to study the feasibility of Gr coatings as MIC resisting elements on non-CVD surfaces, we study the effect of wet-transfer on the quality of graphene. The study revealed that transferred graphene films are significantly more defective than their as-grown counterparts, thereby warranting future research targeting defect-free transfer of graphene for surfaces that are not amenable to CVD growth.

## Methods

### Corrosion Cell

The MIC experiments were carried out in a two-compartment corrosion cell consisting of three-electrodes, i.e. working electrode (nickel anode as a corrosion sample), counter electrode (graphite cathode), and a reference electrode (Ag/AgCl^−^). The cell configuration enables the Ni anode to be immersed in a 0.2 L electrolyte that supports the growth of a diverse set of mixed microbial communities. The Ni anode is coupled to a graphite cathode to promote the bimetallic corrosion. Further, a ferricyanide system (FeCN_6_^3−^/ FeCN_6_^4−^; E^0^ = 0.36 V) with a faster redox kinetics was used as an electron acceptor in the cathode (Comprehensive details in supplementary Fig. S1 & Tables S1, S2, supporting information). The working electrode was a 0.79” × 0.79” nickel (Ni) foam coated with three different materials: i) a Parylene-coated Ni-foam (PA/Ni) ii) a Polyurethane-coated Ni-foam (PU/Ni) and iii) a Graphene-coated Ni foam (Gr/Ni).

### Sample preparation

#### PA/Ni

Clean Ni foam and Si wafer samples (~2 × 2 cm) were placed in a Union Carbide chemical deposition chamber and pumped down to low vacuum conditions of 50 mTorr. A 0.5 g of PA C dimer (Specialty Coating Systems, IN, USA) was sublimed at 150–170 °C and pyrolized at 650 °C. The pyrolized vapors were deposited on the Ni sample at room temperature. A N_2_-based cold trap system was used to remove the unreacted polymer vapors from the deposition chamber. The ellipsometry technique was used to determine the thickness of the PA coating. Light with incident angles of 70° and 75° was reflected on the PA/Ni surface using a VASE Ellipsometer (J.A. Woollam Co., Inc, NE, USA). The observed reflected light was used to determine ratios of the amplitude (ψ) and phase changes of the p and s components of polarized light (Δ)^57^,^58^. A WVASE 32® model was used to minimize mean square error (MSE) and fit the experimental data. X-ray photoelectron spectroscopy (XPS) was then used to confirm the conformal nature of the PA coating (Fig. S6, supporting information).

#### PU/Ni

A Krylon clean polyurethane spray operation was used to establish the polyurethane coating on the Ni foam.

#### Gr/Ni

The chemical vapor deposition (CVD) process was used to grow graphene on the Ni foam. The Ni foam was annealed in H_2_ and Ar atmosphere at 1000 °C to remove the layer of metal oxide. Methane was then introduced to the CVD chamber for 5 mins at ambient pressure. Next, the chamber was rapidly cooled to precipitate the dissolved carbon as a multilayer graphene on the Ni surface^59^.

### Characterization

#### Raman mapping

A Raman microscope (100x objective and 100 μm optical fiber) with a confocal pinhole was used to obtain a Raman mapping of SiO_2_-transferred graphene (80 μm × 80 μm) and CVD-grown graphene (30 μm × 30 μm copper and nickel foam) samples. After data collection, Lorentzian curve fitting was used to fit the D, G and 2D peaks in order to create maps of the spectral features.

#### Scanning electron microscopy

Post experiments, the electrodes were immediately fixed using 2.5% glutaraldehyde buffered with 0.1 M phosphate buffer (pH 7.4). The fixed samples were refrigerated for 24 hours, dehydrated in ethanol series (50%, 70%, 80% and 90%) for 5 minutes at each gradation. The sample was left in 100% ethanol in refrigerator overnight, and dried using an automated critical point dryer (Autosamadri-185, Series A, Tousimis, MD, USA). Post dehydration, a thin layer of Platinum (few nm) was sputtered on the electrode. The samples were then examined in SUPRA® Focused Ion Beam (FIB) under EHT voltage ~1 kV and magnification of 10 kx. The Gr/Ni foam and the transferred Gr samples were viewed without any surface preparation at 3 kV EHT and at low magnifications upto 100x.

#### X-ray photoelectron spectroscopy (XPS)

The Ni foam samples (biotic and abiotic) were analyzed using Al K-alpha X-ray gun at 40W on a Phi Versaprobe® XPS system. After a basic background scan, a detailed scan was conducted with 20 eV pass energy. To confirm the presence of sulfur on a large surface area, a 100 μm × 100 μm sample was chosen with large acquisition time (~75 minutes).

#### Electrochemical testing

Voltage data was acquired periodically using DAQ/54 modules (I/O Tech™, Cleveland OH) across a resistance substitutor (RS 200, IET labs, Inc). Electrochemical impedance spectroscopy was used to analyze the corrosion specimens (i.e. Gr/Ni, PU/Ni, and PA/Ni) with GAMRY ®Reference 3000™. A small AC RMS current (~1 mA) was applied as a perturbation signal in order to measure the impedance response of the anode in a frequency range of 0.1 Hz–10 kHz. The cyclic voltammograms were recorded in the voltage range of −0.8 V–1.0 V and at a scan rate of 25 mV/s.

#### Ni^2+^ measurement

The concentration of free Ni ions (Ni^2+^) in the three corrosion cells were quantified using 1-(2 Pyridylazo)-2-Napthol (PAN) indicator method^60^.

#### Microbial community analysis

Microbial DNA was extracted with PowerSoil® DNA Isolation kit (MoBio) following manufacturer’s guidelines. The 16S rDNA V4 region amplicons (single index) were produced by PCR and sequenced on the MiSeq platform (Illumina) using the 2 × 250 bp protocol yielding pair-end reads that overlap by ~247 bps. Following sequencing, raw BCL files were retrieved from the MiSeq platform and called into fastqs by Casava v1.8.3 (Illumina). The read pairs were demultiplexed based on unique molecular barcodes, filtered for PhiX using Bowtie2 v2.2.1, and reconstituted into two fastq files for each read using standard BASH. A barcodes file was generated from a raw fastq base called previously preserving the original barcode qualities associated per read cluster. Reads were merged using USEARCH v7.0.1001 (allowing 4 mismatches per ≥50 bases) and a barcodes file was generated for the merged set in the same manner they were generated for the raw reads. Processing of sequencing reads included quality-filtering using USEARCH v7.0.1001 (maximum expected error method). Sequences were demultiplexed using QIIME v1.8.0 and then clustered using the UPARSE pipeline. Operational taxonomic unit (OTU) classification was achieved by mapping the UPARSE OTU table to the SILVA database. Abundances were recovered by mapping the demultiplexed reads to the UPARSE OTUs. A custom script constructed an OTU table from the output files generated in the previous two steps. The OTU table was used to calculate alpha-diversity, beta-diversity, provide taxonomic summaries, and in a variety of other analyses built into QIIME that allowed for the characterization of individual and group of samples. Bacterial diversity in each sample was assessed by determining richness based on the calculation of Alpha diversity and Beta diversity in addition to computing diversity indices such as Chao, Simpson, and Shannon.

## Additional Information

**How to cite this article**: Krishnamurthy, A. *et al.* Superiority of Graphene over Polymer Coatings for Prevention of Microbially Induced Corrosion. *Sci. Rep.* **5**, 13858; doi: 10.1038/srep13858 (2015).

## Supplementary Material

Supplementary Information
