# Supplementary material for: Superiority of Graphene over Polymer Coatings for Prevention of Microbially Induced Corrosion
Source: Sci Rep. 2015 Sep 9;5:13858. doi: 10.1038/srep13858 (PMC4563365; doi:10.1038/srep13858)
Supplement: Supplementary Information [file srep13858-s1.pdf]

SUPPORTING INFORMATION

Superiority of Graphene over Polymer Coatings for Prevention of Microbially Induced Corrosion

Ajay Krishnamurthy[1*], Venkataramana Gadhamshetty[2*], Rahul Mukherjee[1], Bharath Natarajan[3], Osman Eksik[1], S. Ali Shojaee[4], Don A. Lucca[4], Wencai Ren[5], Hui-Ming Cheng[5], and Nikhil Koratkar[1, 3]

[1]Mechanical, Aerospace and Nuclear Engineering, Rensselaer Polytechnic Institute, 110 8th Street, Troy, New York 12180, USA

[2]Civil and Environmental Engineering, South Dakota School of Mines and Technology, Rapid City, South Dakota 57701, USA

[3]Department of Materials Sciences and Engineering, Rensselaer Polytechnic Institute, 110 8th Street, Troy, New York 12180, USA

[4]Mechanical and Aerospace Engineering, Oklahoma State University, 218 Engineering North, Stillwater, Oklahoma 74078, USA

[5]Shenyang National Lab for Materials Science, Institute of Metal Research, Chinese Academy of Sciences, Shenyang 110016, China

* Equal Contribution

Correspondence and requests for materials should be addressed to V.G (Venkata.Gadhamshetty@sdsmt.edu) and N.K. (koratn@rpi.edu)

1.0 Corrosion reactor assembly

A detailed drawing of the assembly for a specially configured corrosion cell is shown in Figure S1. Details on the materials used for selected components are shown in Table S1. Two Pyrex® bottles (~500ml) were modified to obtain two identical electrode compartments: i) Anode compartment houses the working electrode and ii) Cathode compartment houses the counter electrode. A glass joint structure (45 mm tube) was integrated on the end of each Pyrex bottle. Each joint has a grove to accept an O-ring that facilitates a tight seal when used with pinch-clamp. A cation exchange membrane was integrated between two compartments before tightening the entire assembly with a pinch-clamp. The bottle cap for each bottle was accommodated with Fisherbrand® rubber septum that carries the titanium wire contact from the working electrode and counter electrode from the two compartments respectively. Both working electrode (WE) and counter electrode (CE) are completely immersed in the respective electrolyte solutions.

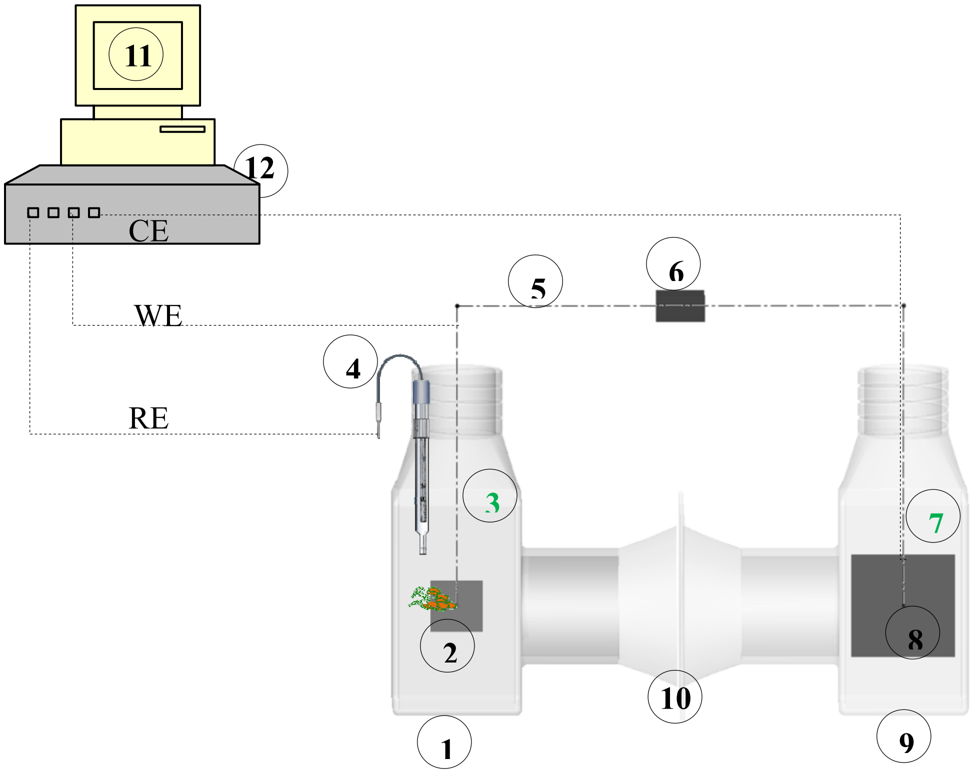

Figure S1. A schematic of the Corrosion Cell. Note: Details of each component in Table S1.

Table S1. Details of the components in corrosion cell.

#	Cell Component	Description	Product Number	Manufacturer Details
1	Anode Compartment	Pyrex Bottle	NC0254567	Fisher
2	Working Electrode	Nickel Foam	INCOFOAMR	Novamet
3	Anolyte Solution	Defined media	NA	NA
4	Reference Electrode	Ag/AgCl-	RREF0021	Pine research instrument[illegible]
5	Titanium Connector	Titanium wire	143-776-31	Good Fellow
6	External Resistor	100 ohm resistance substitutor	RS200	IET Labs
7	Catholyte Solution	Buffered Ferricyanide	702587	Sigma Aldrich
8	Counter Electrode	Carbon Fiber Brush	Panex 35 Carbon Fiber	The Mill Rose Company
9	Cathode Compartment	Pyrex Bottle	NC0254567	Fisher
10	Cation exchange membrane	Nafion 117	274674	Sigma Aldrich
11	Computer	NA	NA	Dell
12	4-channeled Potentiostat	Potentiostat/Galvanostat	®Reference 3000™	Gamry

2.0 Operation details of the microbial corrosion cell

Three identical microbial corrosion cell reactors were operated under a load of 1000Ωs in fed-batch mode supplemented by GSMM. The experimental setups for the MIC experiments have been provided in Table S2. The electrodes chosen for study were suspended in the solution and the inter-electrode spacing was maintained at a bare minimum of 5-8 cm.

Table S2. Experimental setup for the corrosion cells.

	Anode Compartment			Cathode Compartment		
MFC	Anode	Surface Area (m^2)	Anolyte Volume (mL)	Cathode	Surface Area (m^2)	Catholyte Volume (mL)
Ni (parylene)	Nickel + Parylene (46.1nm)	0.004	250	Graphite Brush	1 inch dia and 2 inches length of brush - panex35 carbon fiber	250
Ni (polyurethane)	Nickel + Polyurethane (20-80um)	0.004	250	Graphite Brush	1 inch dia and 2 inches length of brush - panex35 carbon fiber	250
Ni (graphene)	Nickel+Graphene (~1.2nm)	0.004	250	Graphite Brush	1 inch dia and 2 inches length of brush - panex35 carbon fiber	250

The parent microbial reactor was started using the mixed microbial culture inoculated from the wastewater obtained from the Primary Clarifier of the Albany Sewer District. The three microbial corrosion reactors were inoculated using 100ml of the anolyte from the parent microbial reactor. The electrolytes used in the anode and the cathode chambers were prepared using 50mM Phosphate-Buffer Saline (PBS) with the following constituents [g/l] :NH_4Cl, 1.24; KCl, 0.52; $NaH_2PO_4.H_2O$, 2.45; $Na_2HPO_4.7H_2O$, 4.57. The anolyte in addition consists of Sodium 2-Bromoethanesulfonate, 0.4 g/l, glucose, 1.0671 g/l, mineral and vitamin solutions[1] for enriched microbial colonization. The catholyte was spiked with 100mM Potassium Ferricyanide. The anode and the cathode chambers were separated using a Nafion® 117 proton exchange membrane. The Nafion ® 117 membranes are pretreated[2] in order to activate the sulfonic acid sites present in its periphery. The pre-conditioning procedure adopted for the membranes are as follows

1. Boiling for 1hour in De-Ionized water.
2. Boiling in 0.5M H_2SO_4 for one hour.
3. Rinsing in De-Ionized water for removing the excessive acid content.
4. Storing membranes in De-Ionized water for future use.

The cells were kept under a constant load of 1000Ω to promote the growth of anaerobic bacteria and anode-respiring bacteria and introduce galvanic corrosion in the anode. Continuous data acquisition was carried out to monitor the corrosion potential using a DAQ/54module (I/O Tech Inc., Cleveland OH). Regular media replacements were carried out at weekly intervals to ensure nutrient replenishment and prevent excessive Ni accumulation. The Ni concentrations were then estimated from the anolyte media using the 1-(2 Pyridylazo)-2-Naphthol (PAN)[3] method.

3.0 AC electrochemical impedance spectroscopy analysis

The respective circuital parameters for the fit EIS equivalent circuits used for analyzing the Bode plots are given below.

Table S3. The electrical components of the equivalent circuits evaluated for the corrosion cells.

Electrode Type	Rel (kΩ.cm2)	CPE (uF)	Rct (kΩ.cm^2)	Wo (S.s^1/2)	Cdl (μF)	Rint (Ω)	Cm (uF)	Goodness of Fit
Ni (parylene)	0.095312		**0.37776**		256	0.3592	217	0.00673
Ni (polyurethane)	0.075808		**1.5736**	0.01091	42.7	0.26144	337	0.01393
Ni (graphene)	0.07	0.0015	**35.8**	0.003				0.009857

4.0 Raman I(D)/I(G) defect intensity ratios

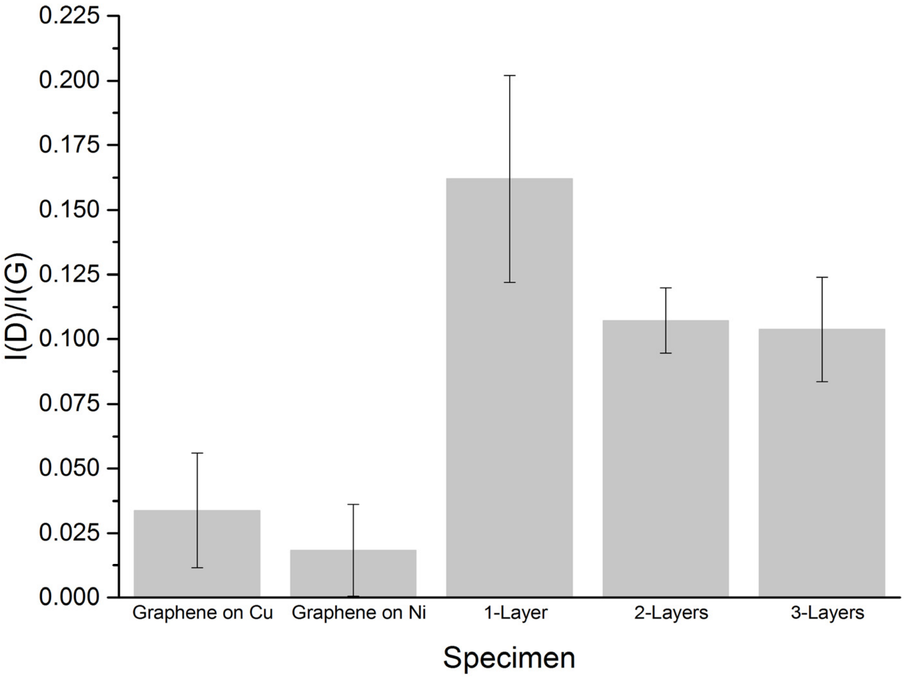

Figure S2. Average Raman defect peak intensities for as-grown 1-layer graphene on Cu and as-grown few-layer graphene on nickel. Data is also shown for 1-layer, 2-layers and 3-layers of graphene transferred from Cu onto SiO_2.

5.0 SEM imaging of transferred graphene samples

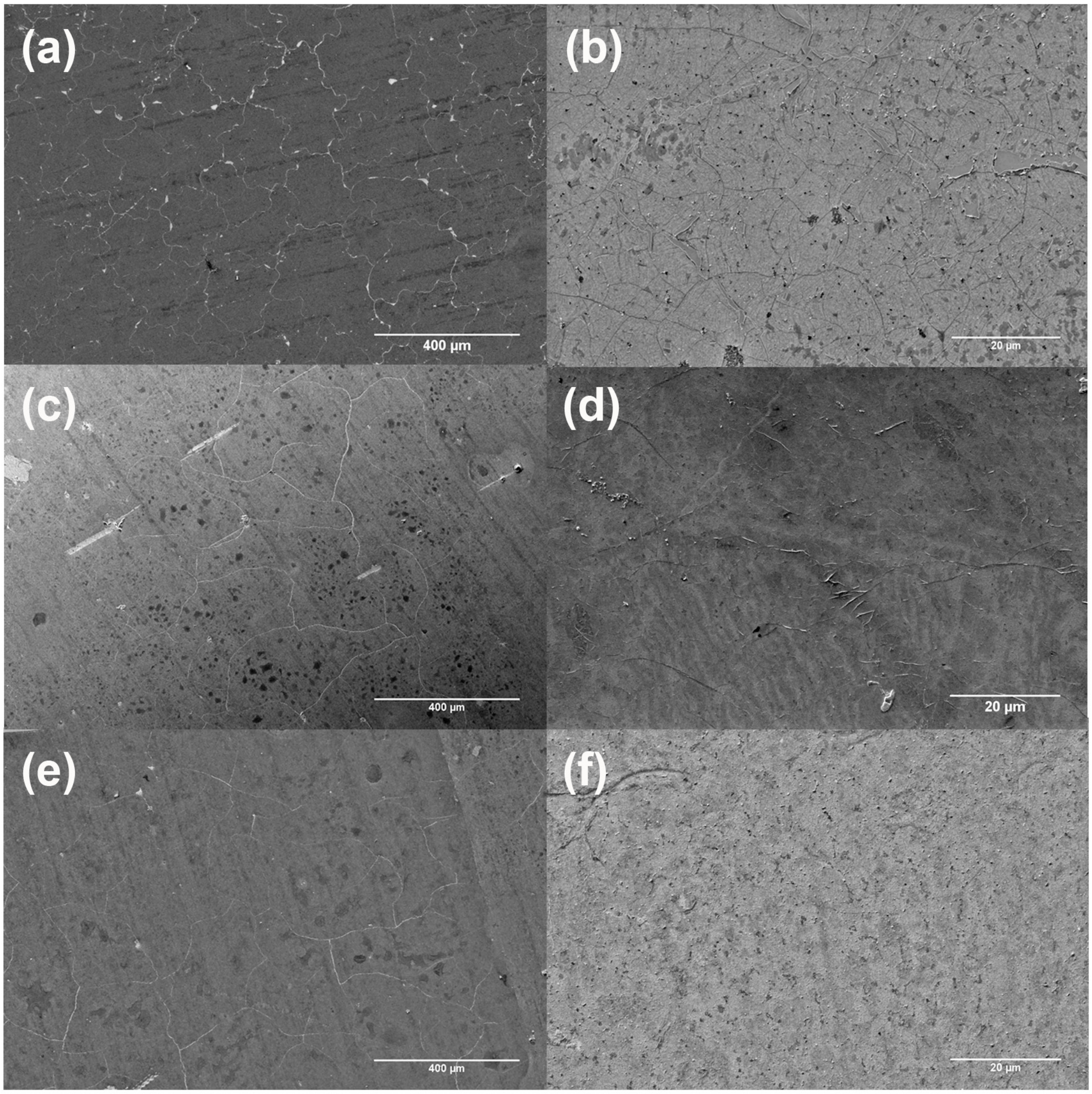

Figure S3. Scanning electron microscopy (SEM) analysis of transferred graphene samples. Monolayer transfer is shown in – (a) low magnification image and (b) high magnification image. Bilayer transfer is shown in (c) low magnification image and (d) high magnification image. Trilayer transfer is shown in (e) low magnification image and (f) high magnification image. In general the extent and severity of the wrinkles tends to decrease from the monolayer to bilayer to trilayer samples.

6.0 Effect of Sulfur reducing bacteria (SRB) on the color of anolyte

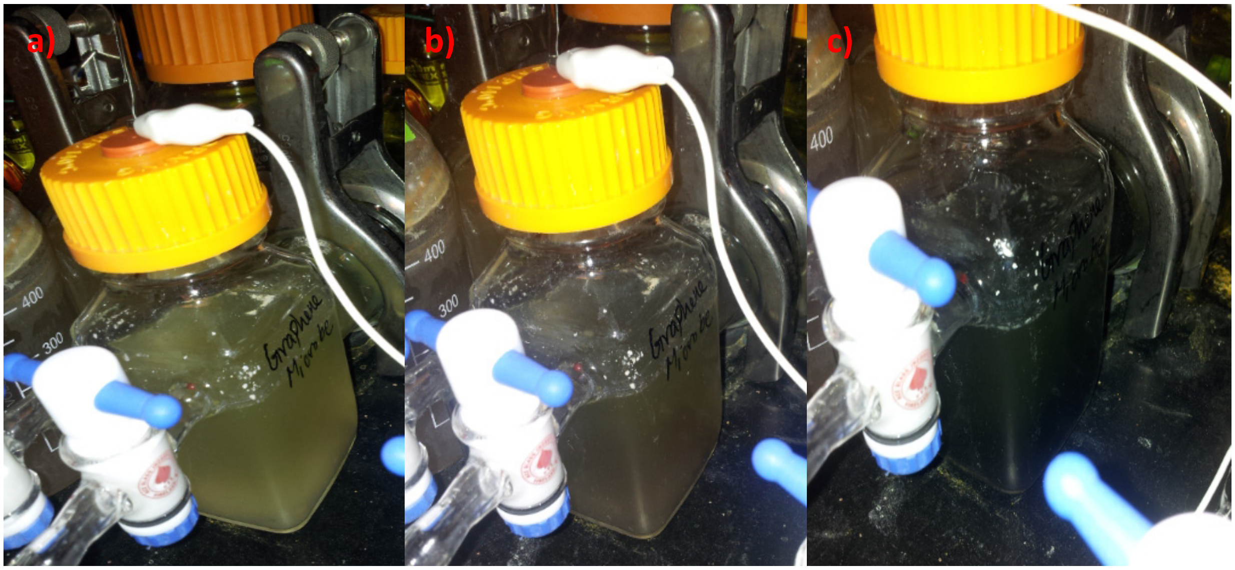

Figure S4. Images of Gr/Ni cell after media replacement, a) immediately, b) ~24 hrs, c) ~48 hrs.

The influence of sulfur reducing bacteria (SRB) on the MIC process is confirmed by the characteristic darkening of the anolyte solution in the anode compartment. Figure S3, shows the progressive color change of media starting from yellow (left, Figure S3a) to greyish-brown close to 24 hrs of cell operation (Figure S3b) and finally completely black after 48 hrs of operation (Figure S3c).

7.0 XPS analysis of biotic and abiotic nickel anodes

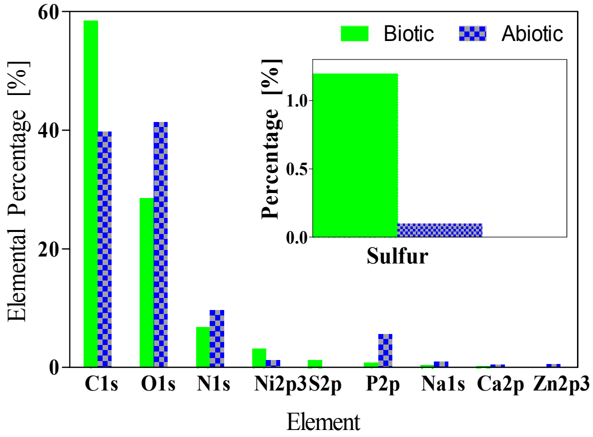

Figure S5. Elemental percentages of corrosion by-products observed on nickel electrodes after exposure to biotic and abiotic environments.

8.0 X-ray photoelectron spectroscopy analysis of PA/Ni electrode

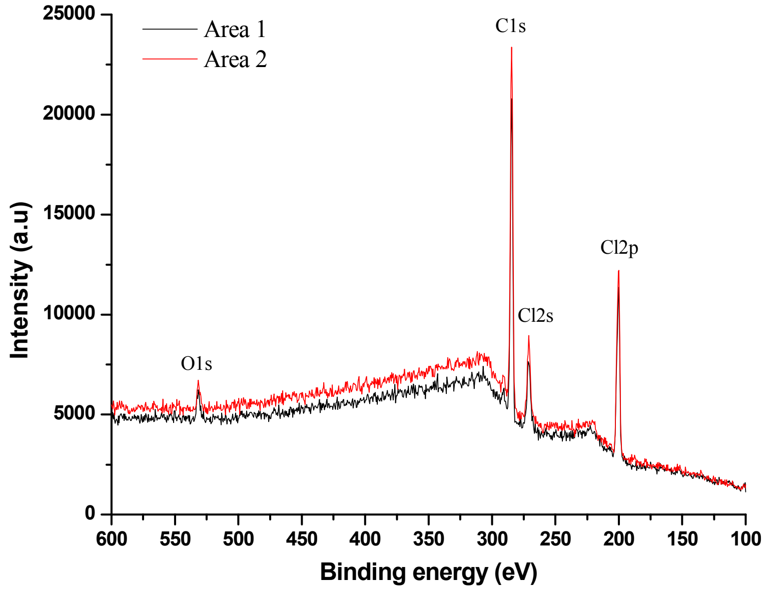

Figure S6. XPS data denoting the presence of parylene C at discrete locations on the PA/Ni sample.

9.0 Supplementary References

1. Cheng, S.; Liu, H.; Logan, B. Increased Power Generation in a Continuous Flow MFC with Advective Flow through the Porous Anode and Reduced Electrode Spacing. *Environ. Sci. Technol.* **2006**, 40, 2426–2432.
2. Lu, Z.; Polizos, G.; Macdonald, D. D.; Manias, E. State of Water in Perfluorosulfonic Ionomer (Nafion 117) Proton Exchange Membranes. *J. Electrochem. Soc.* **2008**, 155, B163.
3. Zarei, K.; Atabati, M.; Malekshabani, Z. Simultaneous Spectrophotometric Determination of Iron, Nickel and Cobalt in Micellar Media by Using Direct Orthogonal Signal Correction-Partial Least Squares Method. *Anal. Chim. Acta* **2006**, 556, 247–254.
